# Telomerase Biogenesis and Activities from the Perspective of Its Direct Interacting Partners

**DOI:** 10.3390/cancers12061679

**Published:** 2020-06-24

**Authors:** Kathryn T. T. T. Nguyen, Judy M. Y. Wong

**Affiliations:** Faculty of Pharmaceutical Sciences, University of British Columbia, Vancouver, BC V6T 1Z3, Canada; kat.nguyen@alumni.ubc.ca

**Keywords:** TERT, TERT interacting proteins, TERT non-telomeric functions

## Abstract

Telomerase reverse transcriptase (TERT)—the catalytic subunit of telomerase—is reactivated in up to 90% of all human cancers. TERT is observed in heterogenous populations of protein complexes, which are dynamically regulated in a cell type- and cell cycle-specific manner. Over the past two decades, in vitro protein–protein interaction detection methods have discovered a number of endogenous TERT binding partners in human cells that are responsible for the biogenesis and functionalization of the telomerase holoenzyme, including the processes of TERT trafficking between subcellular compartments, assembly into telomerase, and catalytic action at telomeres. Additionally, TERT have been found to interact with protein species with no known telomeric functions, suggesting that these complexes may contribute to non-canonical activities of TERT. Here, we survey TERT direct binding partners and discuss their contributions to TERT biogenesis and functions. The goal is to review the comprehensive spectrum of TERT pro-malignant activities, both telomeric and non-telomeric, which may explain the prevalence of its upregulation in cancer.

## 1. Introduction

Telomeres are terminal regions of linear chromosomes, each being a tract of tandem TTAGGG repeats bound by the hexameric protein complex shelterin [1]. The presence of shelterin proteins, consisting of telomeric repeat-binding factor 1 (TRF1, also known as TERF1), telomeric repeat-binding factor 2 (TRF2, also known as TERF2), protection of telomeres protein 1 (POT1), tripeptidyl-peptidase 1 (TPP1, also known as ACD), TRF1-interacting nuclear factor 2 (TIN2, also known as TINF2), and repressor/activator protein 1 homolog (RAP1, also known as TERF2IP), enables the formation of telomere loop (T-loop)—higher-order structure that results from telomeric DNA folding back onto itself—which in turn distinguishes chromosomal ends from DNA breaks that are susceptible to degradation and end-to-end fusion [2,3,4]. Every time a cell divides, telomeric DNA loses 50–100 bp both from its extreme end where the primer of DNA polymerase latches on, and from the resolution of T-loop that allows the replication machinery to pass through [5,6]. When telomeres are eroded to a critical length, either senescence or apoptosis is triggered depending on the cell type [7]. Thus, sufficiently long telomeres are the prerequisite for both genomic integrity and replicative capacity of a cell.

The canonical means of telomere length maintenance is through telomerase—a ribonucleoprotein (RNP) holoenzyme that synthesizes telomeric hexanucleotide repeats from an internal template onto telomeres [8,9]. Telomerase activity is present in embryonic but absent in most adult somatic cells, with the exception of certain populations of tissue stem cells and hematopoietic progenitor cells; thus, telomeres shorten with age by default [10,11]. Age-dependent telomere shortening restricts tissue renewal capacity and represents an important cellular aging mechanism [12]. Accordingly, intrinsically short telomeres secondary to telomerase dysfunction underlie a number of accelerated aging disorders that are collectively referred to as the short telomere syndromes [12,13]. On the other hand, since telomere length dictates cell division potential, overriding the endogenous telomere attrition process is crucial for tumorigenesis [1]. While telomerase-independent strategies are available, the majority of human cancers (85–90%) employ telomerase to maintain the length of their telomeres above the senescence-inducing threshold [14].

**Figure 1:**
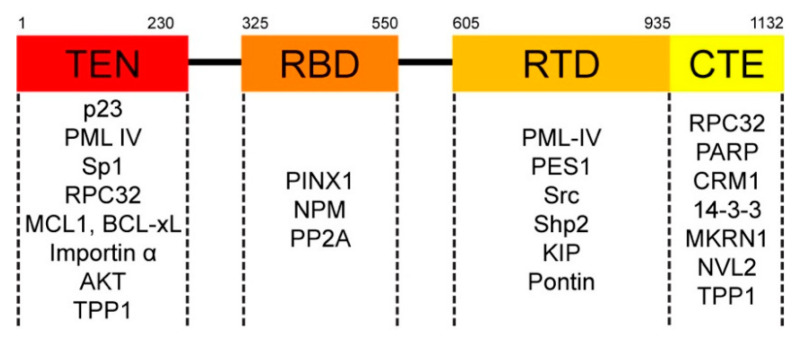
2D schematic of the full-length telomerase reverse transcriptase (TERT) protein, which consists of the telomerase essential N-terminal (TEN) domain, the RNA binding domain (RBD), the reverse transcriptase domain (RTD), and the C-terminal extension (CTE) domain. The binding partners interacting with each domain of TERT are specified.

The catalytic core of telomerase is made up of telomerase RNA component (TERC) and telomerase reverse transcriptase (TERT) [15]. The 5’ end of TERC bears the telomeric repeat template, whereas the 3’ end contains a hairpin-hinge-hairpin-ACA (H/ACA) motif that mediates TERC interaction with two sets of the H/ACA-RNP complex, each of which consists of dyskerin (also known as DKC1, NAP57, or Cbf5p), NHP2, NOP10, and GAR1 [16,17]. The H/ACA-RNP complexes protect TERC from exonucleolytic degradation, and they remain bound to TERC as part of the fully assembled telomerase holoenzyme [15]. On the other hand, the full-length TERT protein (FL TERT) consists of four main functional regions (Figure 1). The N-terminus comprises the telomerase essential N-terminal (TEN) domain, which tethers to telomeres during catalysis, and the RNA binding domain (RBD) that directly associates with TERC [16]. Between the two termini is the reverse transcriptase domain (RTD)—the catalytic site of telomerase [18]. The C-terminus of TERT (CTE) does not have a precisely defined function; however, mutations within this region have been shown to impair both subcellular localization and activity of telomerase [16].

Human TERC and TERT are encoded by *hTERC* on chromosome 3 and *hTERT* on chromosome 5, respectively [16]. While *hTERC* expression is ubiquitous, *hTERT* expression is largely suppressed in adult somatic cells; thus, with TERT being the rate-limiting determinant of telomerase activity, its transcriptional upregulation is an expected hallmark of telomerase-positive tumors [19]. However, given that the amount of telomerase activity a cell needs to overcome senescence corresponds to as low as 1% of TERT expression level in cancer [20,21], TERT likely has pro-malignant functions that are independent from its canonical role in telomere extension. Indeed, evidence of TERT non-telomeric activities—regulation of apoptosis, DNA damage response, and transcription among others—in the context of cancer has amassed since the late 1990s [21]. Furthermore, given that expression of certain TERT alternative splice variants whose products are catalytically inactive proteins can enhance survival and promote cell proliferation post-DNA damage, TERT non-telomeric functions can be uncoupled from the canonical telomerase activity [22,23].

In vitro protein-protein interaction (PPI) assays including yeast two-hybrid assay, affinity and gel filtration purification have discovered numerous species that directly interact with TERT at different subcellular compartments. However, while the TER-associated H/ACA-RNP complexes are stable components of the telomerase holoenzyme, many of TERT binding partners make transient association within subpopulations of TERT complexes (Table 1). In the following sections, we discuss the dynamic regulation of these TERT interacting proteins and their known contributions to telomerase biogenesis and activity, as well as to TERT non-telomeric, pro-malignant functions.

## 2. TERT Protein Interactions Implicated in Its Trafficking between the Cytoplasm and the Nucleus

**Figure 2:**
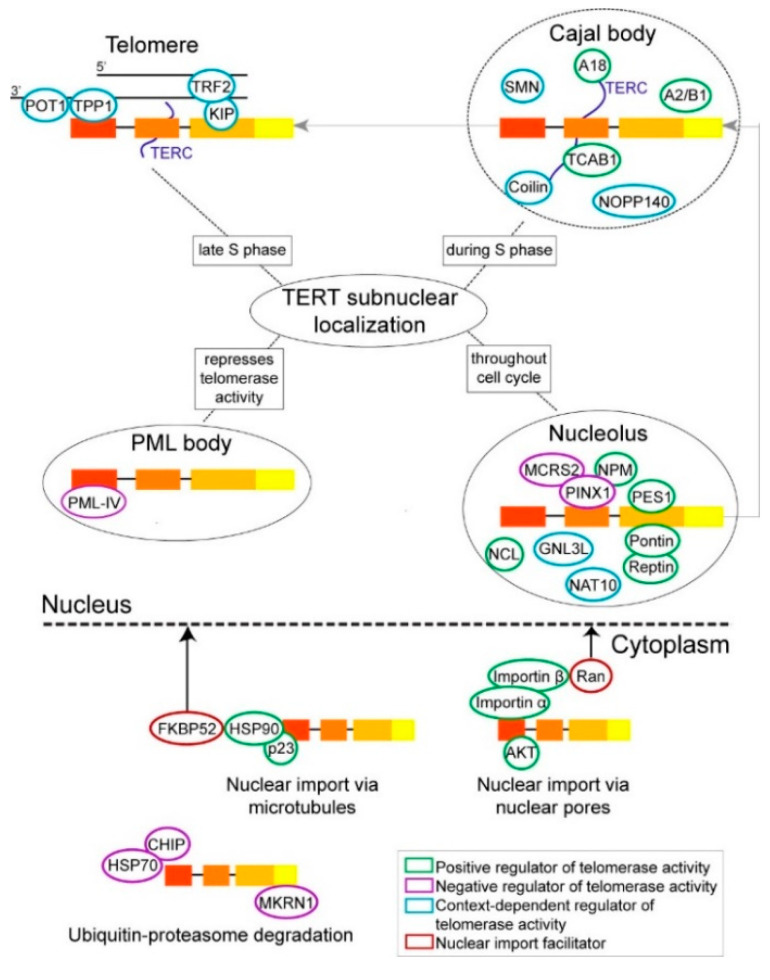
TERT interacting partners involved in telomerase catalytic function at telomeres. The proteins are placed in the subcellular compartment where they are reported to primarily co-localize with TERT. To highlight the dynamic changes in TERT interacting partners outside of telomerase assembly, components of the telomerase holoenzyme, such as H/ACA proteins dyskerin, NHP2, NOP10, and GAR1, are collectively represented as TERC (dark blue). Species whose binding sites on TERT have not been mapped are not placed directly onto TERT. (**Cytoplasm**) The most established mechanisms of TERT nuclear import and cytoplasmic retention are illustrated. (**Nucleus**) TERT localization in the nucleolus, Cajal bodies, and at telomeres are related to H/ACA the cell cycle-dependent dynamics of telomerase assembly and catalytic action. TERT localization in promyelocytic leukemia (PML) bodies reflects retention in the nucleoplasm, which limits the pool of TERT molecules available for telomerase assembly and activity.

Following peptide synthesis in the cytoplasm, TERT typically translocates into the nucleus where its telomeric function takes place. Multiple cellular mechanisms are known to facilitate TERT nuclear localization (Figure 2). The heteromeric complex consisting of heat shock protein 90 (HSP90), its co-chaperone p23, and FK506-binding protein 52 (FKBP52, also known as peptidyl-prolyl cis-trans isomerase FKBP4) was reported to bring TERT into the nucleus along microtubules in H1299 lung cancer cells, by virtue of the interaction between FKBP52 and the dynein/dynactin motor [24,25]. Additionally, HSP90 binding enables TERT phosphorylation by AKT—a serine/threonine-specific protein kinase, which in turn enhances telomerase activity [26,27]. Conversely, serine/threonine-protein phosphatase 2A (PP2A) can dephosphorylate both AKT and TERT whose interaction is stabilized by HSP90, thus, in effect abrogating telomerase activity [27]. Separately, the importin system was found to mediate TERT nuclear import in MCF7 breast cancer cells; in particular, importin α binds TERT N-terminal nuclear localization signal (NLS) while its co-adaptor importin β1 binds Ran—a GTPase that shuttles its substrates in and out of the nucleus across the nuclear pore [28]. Furthermore, AKT-mediated phosphorylation at residue S227 enhances TERT affinity for importin α, corresponding to an increase in TERT nuclear localization [28]. Taken together, this finding and the observation that PP2A overexpression increases TERT presence in the cytoplasm [29] indicate that post-translational modification may directly influence TERT subcellular distribution. Lastly, the Tsuruo lab discovered in 2000 that 14-3-3 binding site on TERT is the C-terminal amphipathic helix directly downstream of the leucine-rich nuclear export signal (NES); thus, they postulated that 14-3-3 promotes TERT nuclear localization by preventing the binding of chromosome region maintenance 1 protein homolog (CRM1, also known as exportin-1) to the NES motif [30]. The 14-3-3—TERT interaction itself may be PP2A-mediated, as the amount of 14-3-3 co-purified with TERT is significantly less in HeLa cells overexpressing PP2A compared to parental cells [29]. However, whether 14-3-3 is essential to TERT nuclear localization is controversial. While the Tsuruo lab found that TERT bearing a triple mutation in the 14-3-3 binding site (14-3-3mt TERT) accumulates in the cytoplasm of HEK293T cells [30], the Mattson lab reported that the same TERT mutant still resides primarily in the nucleus of HeLa cells [31]. Nevertheless, given that depletion of either HSP90, FKBP52 or importin α significantly impairs telomerase activity [24,25,28], whereas 14-3-3mt TERT and FL TERT have comparable telomerase activity [30], the HSP90—FKBP52 and importin mechanisms are likely the primary facilitators of TERT nuclear translocation and function. Because the two systems have only been assessed separately, to what extent each contributes to TERT nuclear import in the same cellular context remains unknown.

Apart from its role in TERT nuclear import, HSP90 also protects TERT from degradation outside of the nucleus. As TERT sensitivity to HSP90 antagonist geldanamycin can be rescued by pre-treatment with specific proteasome inhibitor MG132 but not lysosomal proteolysis inhibitor E64 [32], TERT is specifically vulnerable to ubiquitin-proteasome degradation. While HSP90 presence shields TERT from the ubiquitin ligase E3 ubiquitin-protein ligase makorin-1 (MKRN1) [32], its close relative heat shock protein 70 (HSP70) promotes MKRN1 activity upon binding to TERT, since CHIP (Carboxyl-terminus of HSP70 Interacting Protein)—the co-chaperone of HSP70—is capable of displacing p23 from HSP90, which in turn destabilizes the TERT-HSP90 complex [33]. Collectively, the two heat shock proteins and their co-chaperones work in concert to modulate TERT stability in a cell cycle-dependent manner, given that formation of the HSP70-CHIP-TERT complex is restricted to the G2/M phase [33]. Interestingly, though HSP70-TERT binding endorses TERT degradation, a recent study demonstrated that the same interaction facilitates HSP70 co-localization at telomeres with the telomere protective protein Apollo, corresponding to a decrease in the number of telomere dysfunction-induced foci [34]. This finding suggests that TERT contribution to telomere maintenance is not limited to the de novo synthesis of telomeric repeats. In fact, the possibility of telomerase binding to telomeres as a protective cap has been raised by the Blackburn lab in 2003, when they observed non-homologous fusion between critically short telomeres in telomerase-deficient Saccharomyces cerevisiae cells [35].

As previously mentioned, TERT can interact with CRM1 via its C-terminal NES motif. Upon CRM1 binding to Ran, the entire complex moves out of the nucleus via the nuclear pore [36]. Efficient TERT nuclear export requires the phosphorylation of residue Y707 by tyrosine kinase Src, whereas dephosphorylation of the same residue by tyrosine phosphatase Shp2 retains TERT in the nucleus [36,37]. Both exo- and endogenous oxidative stress can stimulate TERT cytoplasmic translocation, and by virtue of its N-terminal mitochondria-targeting sequence, TERT subsequently shuttles to the mitochondrial matrix, most likely in the company of a chaperone protein that has yet been identified [36,38]. However, the biological effect of TERT’s non-canonical activity at mitochondria remains controversial to date. While TERT has been shown to protect mitochondrial DNA integrity and function from H_2_O_2_-induced damage in human umbilical vein endothelial cells (HUVECs) [39], it apparently exacerbates the same damage in normal human fibroblasts [38]. Similarly, inhibition of TERT nuclear export promotes survival of HEK293T cells post-oxidative stress [36], whereas HeLa, MCF7, and U87 cells in which TERT mitochondrial localization is enabled are more resistant to apoptosis following the same treatment [40]. Such disparity suggests that TERT mitochondrial function may be context-dependent and/or cell-type specific.

TERT dynamic subcellular localization is particularly crucial in the context of cancer, as evidenced by the observation that TERT bearing mutations in the NES motif cannot immortalize cells despite intact catalytic activity [41]. Furthermore, expression of these mutant proteins sensitizes cells to genotoxic stress, as well as induces cell cycle arrest [42]. Taken together, these findings indicate that targeting TERT intracellular trafficking is a viable anti-cancer approach.

## 3. TERT Protein Interactions Implicated in Its Trafficking within the Nucleus

While mature TERC primarily accumulates in Cajal bodies (CBs)—membraneless subnuclear structures in which small nuclear RNP (snRNP) assembly takes place—by virtue of the Cajal body localization box (CAB box) motif at its 3’ end being the binding site of the CB-specific chaperone telomerase Cajal body protein 1 (TCAB1) [43,44,45], trafficking of TERT within the nucleus is cell cycle-sensitive (Figure 2). It is detected in the nucleolus—the site of ribosome biogenesis—through all phases of the cell cycle, and in CBs only during S phase [46,47]. However, whereas TERT can exist independently from TERC in the nucleolus, as more than one nucleolar localization signal motifs have been identified on TERT [48,49,50], TERT presence in CBs depends on its TERC-mediated interaction with TCAB1 [51]. Reciprocally, TERC accumulation in CBs was found to be exclusive to TERT-positive cells [52]. Given that (1) TERC and the CB marker coilin are detected at the nucleolus periphery in early S phase, (2) TERT CB localization peaks in late S phase, and (3) CBs are specific to transformed cells, the nucleolus is likely the primary site of telomerase assembly while the motile CBs function as a delivery system, first depositing TERC at the nucleolus for complex formation with TERT, then uptaking and bringing the fully assembled holoenzyme to telomeric DNA [53,54,55]. That CBs facilitate telomerase recruitment to telomeres is elegantly demonstrated by the 2016 live single-cell imaging study by the Cech lab. Using CRISPR to tag endogenous TERT and TRF2—a shelterin component—of HeLa cells with fluorescent labels, the investigators discovered that telomerase-containing CBs diffuse in a three-dimensional pattern through the nucleus during S phase, forming both short-dynamic and long-static interactions with telomeres [56]. Additionally, TERT can be retained in the nucleoplasm as a result of its binding to promyelocytic leukemia (PML) protein isoform 4 (PML IV)—a constituent of distinct structures that bind tightly to the nuclear matrix known as PML bodies [57]. The finding that telomerase activity in PML-knockdown (KD) H1299 cells is two-fold of that in parental cells [58] implies that telomerase activity is partly determined by the relative distribution of TERT between different subnuclear compartments.

### 3.1. TERT Protein Interactions in the Nucleolus

Of the nucleolar TERT-interacting species, the tumor suppressor PIN2/TERF1-interacting telomerase inhibitor 1 (PINX1) is an established negative regulator of telomerase activity [59]. While PINX1 N-terminus also interact with TERT, the telomerase inhibitory domain (TID) is within the C-terminus [59]. On the other hand, PINX1 binding site is mapped to the RBD domain of TERT [60]. However, given that TERC still co-purifies with TERT in PINX1 presence, PINX1 may not interfere with telomerase assembly [60]. Since PINX1 also interacts with the shelterin protein TRF1 [59], PINX1 likely interferes with telomerase catalytic action at telomeres. Recently, nucleophosmin (NPM)—another nucleolar species—was found in the same complex with PINX1 and TERT [61]. Given that NPM binding occurs at PINX1 C-terminus, and that NPM addition rescues telomerase activity in cells expressing PINX1, it has been proposed that NPM attenuates PINX1 inhibitory effect on telomerase by replacing TERT at PINX1 TID [61]. Additionally, the formation of the PINX1-NPM-TERT heterotrimer is S phase-specific, peaking at early S phase in nucleolar foci [62]. Taken together, these findings indicate that PINX1 regulates TERT availability in a cell cycle-dependent manner, with NPM joining the complex when the need for telomerase activity arises.

The second nucleolar negative regulator of telomerase activity is microspherule protein 2 (MCRS2), which was first discovered as a PINX1-binding protein, co-localizing with PINX1 both in the nucleolus and at telomeres [63]. However, while PINX1 expression is detected at all phases of the cell cycle, MCRS2 expression is restricted to the early S phase [63]. Additionally, although MCRS2 co-purified with TERT in HEK293T cells, it is not clear whether the interaction is independent of PINX1 [63]. It is possible that MCRS2 and NPM are both S phase-specific PINX1 co-effectors, working against each other to modulate the pool of TERT available for telomerase assembly.

Nucleolin (NCL) is a phosphoprotein found primarily in the dense fibrillar compartment of the nucleolus [64]. It has two distinct TERT binding sites—one that facilitates direct protein-protein interaction and one that is TERC-mediated, as evidenced by RNase A treatment reducing but not eliminating TERT-NCL interaction in vitro [65]. Given that NCL overexpression has little effect on telomerase activity, NCL may not participate in functionalizing the holoenzyme [65]. Interestingly, co-expression of NCL and TERT in VA13 cells which endogenously lack TERT results in TERT nucleolar enrichment [65], suggesting that NCL facilitates TERT nucleolar localization.

Three ATPases—pontin, reptin, and nuclear valosin-containing protein-like 2 (NVL2)—are among nucleolar proteins reported to associate with TERT. Pontin and reptin often bind to each other, forming a dimer [66]. As pontin can bind to both TERT RTD and dyskerin—a member of the TERC-associated H/ACA-RNP complex, the pontin/reptin dimer may enable telomerase assembly by bringing the two subunits together [67]. Although pontin and TERC do not co-purify, pontin depletion impaired TERC accumulation, suggesting that pontin ATPase activity is necessary for maintaining the endogenous TERC level [67]. Additionally, while the amount of dyskerin bound to pontin/reptin remains largely unvaried between the phases of the cell cycle, TERT-pontin/reptin complex formation in S phase was found to be three-fold of that in G2, M, or G1 [67], suggesting that pontin/reptin promotes TERT availability for telomerase assembly. On the other hand, NVL2 immunoprecipitate from HeLa cells exhibits telomerase activity [68], suggesting that NVL2 also interacts with the catalytically active holoenzyme. Importantly, mutations in NVL2 N-terminal ATP-binding domain impair both NVL2-TERT interaction and telomerase activity [68], which indicates that NVL2 contribution to telomerase assembly involves its ATPase function.

Two additional NTPases—N-acetyltransferase 10 (NAT10, also known as RNA cytidine acetyltransferase) and guanine nucleotide-binding protein-like 3-like protein (GNL3L)—are also identified as TERT binding partners in the nucleolus [69]. NAT10 readily interacts with TERT in the absence of TERC, and only co-enriches TERC in the presence of TERT, suggesting that the TERT-NAT10 association is via direct protein-protein contact [69]. Since GNL3L co-purifies with even less TERC than NAT10, the TERT-GNL3L complex may also be TERC-independent [69]. The observation that dyskerin immunoprecitate exhibits less telomerase activity than either NAT10 or GNL3L indicates that the two NTPases interact preferentially with the catalytically active telomerase holoenzyme [69]. Curiously, while NAT10 overexpression stimulates a 2.5-fold increase in telomerase activity in cell extracts, overexpression of either NAT10 or GNL3L induces progressive telomere shortening in intact cells [69]. It is possible that the two NTPases dynamically regulate the assembly and disassembly of the telomerase holoenzyme in a context- and/or cell cycle-dependent manner.

Pescadillo homolog (PES1) is the latest nucleolar protein identified as a TERT-binding species. PES1 interaction with TERT is not sensitive to RNase A treatment, and thus, PES1 makes contact with TERT via direct protein-protein binding [70]. Moreover, since it co-purifies with TERC and dyskerin in the presence of TERT, and its expression correlates positively with telomerase activity both in vitro and in clinical breast cancer samples [70], PES1 may make contact with the telomerase RNP. Curiously, PES1 does not co-purify with other known TERT-interacting proteins—HSP90, p23, and fellow nucleolus residents pontin and reptin—in MCF7 cells [70]. As PES1 binds TERT at similar levels throughout the cell cycle [70], whereas pontin/reptin association with TERT specifically peaks in S phase [67], PES1 not immunoprecipitating pontin/reptin may reflect a temporal exclusivity that could not be captured by the PPI method in use. In addition to telomerase activity, PES1 expression was found to promote the proliferation of MCF7 cells in a TERT-dependent manner, which also corresponds to c-MYC upregulation [70]. Nevertheless, because the TERT-PES1 interaction was discovered recently and has been assessed only in the context of breast cancer cells, follow-up studies are required to fully characterize PES1 role in telomerase biogenesis and functions.

### 3.2. TERT Protein Interactions in the Cajal Bodies

In CBs, TERT interacts with several local species, including coilin—the scaffold protein that upholds CB structure [51]—and survival motor neuron protein (SMN). Coilin interaction with TERT is TERC-mediated, while SMN binds TERT directly [71,72]. However, the regulatory effect of either coilin or SMN on telomerase activity is still poorly understood. Coilin depletion in HEK293T cells does not significantly reduce telomerase activity [51], whereas SMN immunoprecipitate telomerase activity in the same cell line [72]. On the other hand, a study using HeLa cells found that overexpression of either coilin or SMN minimally disrupts the composition of the TERC—H/ACA complex and has no effect on telomerase activity [73]. It is possible that coilin and SMN implications in telomerase biogenesis are cellular transformation state- and/or cell type-specific.

Heterogeneous nuclear ribonucleoproteins (hnRNPs) are a family of RNA-binding proteins that participate in mRNA biogenesis in the nucleus and its subsequent translation in the cytoplasm [74]. Of the several hnRNPs that are involved in telomere and telomerase biology [75], two specifically co-localize with TERT in CBs: A2/B1 and A18. A2/B1 and TERT co-purify both in vitro and in hepatocellular carcinoma (HCC) tissue samples, and A2/B1 depletion impairs telomerase activity in HCC HUH7 cells [76]. A2*, an A2 splice variant, can simultaneously bind single-stranded telomeric DNA and TERT, and its ability to dissolve telomeric G-quadruplex enhances telomerase activity in vitro [77]. On the other hand, the interaction between A18 and TERT is vulnerable to RNase A treatment and thus is TERC-mediated. As A18 depletion reduces the amount of TERT mRNAs in vitro, A18 may regulate telomerase activity at the gene expression level as well [78].

As mentioned previously, CBs participate in delivering telomerase to telomeres by virtue of their motility [53]. However, whether telomerase localization in CBs is obligatory for telomere catalysis is being actively debated. In 2012, the Bryan lab observed (1) the rescue of telomerase activity by TERT overexpression, and (2) the co-localization of TCAB1 and TERT at telomeric foci in coilin-KD HEK293T cells that lack CBs [79]. The investigators thus postulated that TERT localization in CBs depends on its own expression level, and that TCAB1 is capable of driving telomerase to telomeres in a CB-independent manner. In 2016, the Collins lab found that both TCAB1 and CBs are dispensable for telomere maintenance in HCT116 and U2OS cancer cells, and in VA13 immortalized fibroblasts [80]. Most recently, the Meier lab reported that HeLa cells depleted of NOPP140—an intrinsically unstructured phosphoprotein that interacts with the TERC-associated protein dyskerin in both the nucleolus and CBs [81,82]—exhibit drastic redistribution of telomerase from CBs to the nucleoplasm, which corresponds to a slow rate of telomere lengthening over 400 population doublings [83]. This latest finding supports the Collins lab’s proposal that CBs represent an efficient but non-obligatory means to shuttle telomerase to telomeres. Nevertheless, further investigation is needed to better understand the implication of CB localization in regulating telomerase activity.

### 3.3. TERT Protein Interactions at the Telomeres

TPP1 and TRF2 are the two shelterin proteins responsible for directly tethering telomerase to its substrate—the single-stranded G-rich overhang at the 3’ end of telomeric DNA (ssDNA_tel_). The oligonucleotide/oligosaccharide binding (OB) domain of TPP1 contains the TEL (TPP1 glutamate (E) and leucine (L)-rich) patch and a region in its N-terminus that respectively engage the TEN and CTE domains of TERT [84,85]. On the other hand, TRF2–TERT association is mediated by KIP (also known as calcium and integrin-binding family member 1, or CIB1) [86,87]—a binding partner of the non-homologous end-joining (NHEJ) DNA damage repair protein DNA-dependent protein kinase catalytic subunit (DNA-PKcs) [88]. Considering that TPP1 depletion correlates with a significant loss of TERT foci at telomeres [84], whereas KIP-KD has negligible effect on either telomerase activity or telomere length [86], TPP1 is more essential to telomerase recruitment than TRF2. As TPP1 is unstable on its own, it forms a heterodimer with the fellow shelterin subunit POT1, which binds exclusively to ssDNA_tel_ [89]. As POT1 is capable of resolving G-quadruplexes on ssDNA_tel_, the efficiency of telomerase processivity is POT1-dependent [90]. The observations that the expression of POT1 mutants with defective DNA-binding or TPP1-binding domains results in longer but fragile telomeres in vitro [89,91] indicate that the TPP1-POT1 dimer fine-tunes telomerase activity to maintain telomere homeostasis. In corroboration, the Lingner lab demonstrated in 2012 that the human CTC1-STN1-TEN1 (CST) complex terminates telomerase activity in late S/G2 phase by competing with TPP1-POT1 for ssDNA_tel_ binding [92], highlighting the role of TPP1-POT1 in regulating telomerase activity. Most recently, the Taylor lab proposed that ssDNA_tel_ length prescribes TPP1-POT1 regulatory effect on telomerase, as they found that the level of telomerase activity is inversely proportional to the length of ssDNA_tel_ in complex with TPP1 and POT1 [93]. Additionally, the 2017 study by the Cech lab reported a novel TIN2-TRF2-TPP1 complex that lacks POT1, which stimulates a level of telomerase activity equivalent to that of the TPP1-POT1 dimer [94]. However, given that both TIN2 and TRF2 are double-stranded DNA-binding proteins, the TIN2-TRF2-TPP1 trimer may only be significant to telomerase recruitment at critically short telomeres.

Ku—another component of the NHEJ pathway—is a heterodimeric complex made up of two evolutionarily conserved proteins Ku70 and Ku80 [95]. While Ku participation in telomere maintenance is well-characterized in Saccharomyces cerevisiae, less is known about its telomeric function in humans, although Ku has been found in complex with both human TERT and TERC independent of DNA-PKcs [96,97]. Interestingly, a 2012 study found that in Saccharomyces cerevisiae, Ku-DNA and -RNA interactions are mutually exclusive, and accordingly, Ku is incapable of tethering telomerase to telomeric DNA [98]. The authors thus propose a “hand-off” model, in which Ku recruits then passes telomerase to the shelterin proteins. Whether this model holds true in humans requires further investigation.

## 4. TERT Protein Interactions and Non-Telomeric Activities in the Context of Cancer

**Figure 3:**
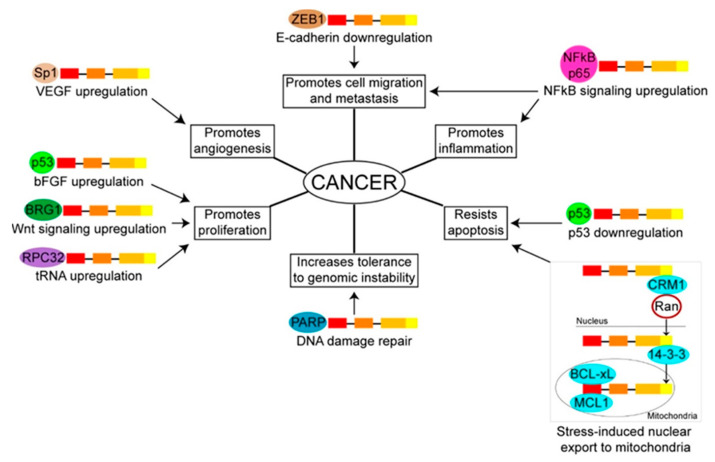
TERT interacting partners and their proposed contributions to TERT non-telomeric activities in the context of cancer. The pro-malignant effect that each TERT protein interaction induces is specified.

Increased tolerance to DNA damage and anti-apoptotic signaling are among the most documented non-telomeric functions of TERT. Several nuclear TERT interacting proteins contribute directly to these activities, including p53, poly [ADP-ribose] polymerase (PARP), and 14-3-3 (Figure 3). The observations that p53 upregulation rescues, whereas p53 downregulation exacerbates, TERT depletion-induced apoptosis were initial evidence of p53 mediating TERT pro-survival effect [99]. In 2010, the Kim lab found that upon doxorubicin-induced DNA damage, recombinant TERT simultaneously suppresses p53 activation and promotes basic fibroblast growth factor (bFGF) expression, thus in effect enhancing survival and subsequent proliferation of both immortalized human fetal fibroblasts and cancer cells in vitro [100]. Interestingly, FL TERT and the D712A TERT mutant, which carries a defective RTD domain and thus is catalytically inactive, produce similar levels of bFGF expression [100], indicating that TERT p53/bFGF-mediated anti-apoptosis activity is independent from the canonical telomeric function. Additionally, p53 and TERT were reported to form a heteromeric complex with the DNA damage repair protein PARP [99], suggesting that TERT may participate in DNA damage repair. Given that PARP interacts with TERT at the latter’s NES motif [99,101], it is also possible that PARP dictates nuclear retention of TERT when DNA damage occurs. 14-3-3, which was discussed earlier (Section 2), may also take part in TERT anti-apoptosis function, since the expression of 14-3-3mt TERT correlates with decreased resistance to staurosporine- and etoposide-induced apoptosis in HeLa cells [31].

TERT anti-apoptotic property may extend beyond the nucleus. As aforementioned (Section 2), TERT re-enters the cytoplasm upon oxidative stress and travels to the mitochondria, which houses a distinct pool of apoptosis-related proteins. That expression of 14-3-3mt TERT correlates with greater release of cytochrome c and apoptosis-inducing factor in GM847 cells compared to FL TERT [31] which indicates that part of TERT apoptotic function occurs in the mitochondria, and that 14-3-3, despite its debatable role in TERT nuclear localization, may facilitate TERT mitochondrial translocation. To date, the only mitochondrial species confirmed to be TERT interacting partners are the anti-apoptotic proteins induced myeloid leukemia cell differentiation protein MCL1 (MCL1) and BCL2-associated agonist of cell death (BCL-xL), both of which bind to the BH3-like motif in TEN domain of TERT [102]. However, the exact significance of TERT association with these species requires follow-up studies, for within the scope of the initial report, the investigators found that TERT expression did not significantly enhance nor attenuate MCL1 or BCL-xL activities.

Transcription regulation is another major non-telomeric function of TERT that involves a number of its interacting partners (Figure 3). In their 2009 study using mouse embryonic stem cells, the Artandi lab demonstrated an interaction between TERT and BRG1—an upstream component of the pro-proliferative Wnt signaling pathway [103]. The TERT-BRG1 complex in turn promotes transcription of Wnt-dependent genes, including c-MYC and cyclin D1, by physically occupying their promoter regions [103]. As c-MYC is known to upregulate TERT expression [104], TERT interaction with BRG1 may create a positive feedback loop that co-enhances telomerase activity and cell proliferation, both of which are beneficial to tumor development. However, TERT role in Wnt signaling is a controversial subject. In 2011, the Greider lab found TERT depletion having no effect on the expression level of genes in the Wnt pathway; thus, they contended that TERT overexpression in the original report by the Artandi lab might have produced physiologically irrelevant results [105]. On the other hand, given the observations that endogenous Wnt signaling is unproportional to telomerase activity level, and that TERT overexpression hyperactivates the Wnt pathway in only one out of four breast cancer cell lines, the Blackburn lab proposed in 2014 that TERT participation in Wnt signaling is highly context-dependent [106]. While the significance of TERT—BRG1 association in Wnt signaling remains to be further investigated, the heterotrimeric complex consisting of TERT, BRG1, and the nucleolar protein nucleostemin—a GNL3L (Section 3.1) paralog that likely does not participate in TERT catalytic action at telomeres [69]—has been studied extensively, with implicated non-telomeric functions including tumor initiation, heterochromatin assembly, and microRNA regulation [107,108,109].

TERT can participate in the NFκB signaling pathway as well, given that depletion of the NFκB p65 subunit attenuates TERT pro-survival and pro-proliferative effects in vitro [110]. Upon induction by TNFα—a pro-inflammatory factor, TERT and the NFκB p65 subunit were found to physically interact at the promoter region of selective NFκB target genes, including IL6, IL8, TNFα, and the *hTERT* gene itself, whose expression levels are consequently upregulated [110,111]. In effect, the interaction between TERT and NFκB p65 may also present cancer cells with a pro-malignant feedback loop that simultaneously sustains cell proliferation, chronic inflammation, and telomerase activity. Additionally, TERT has been shown to upregulate the expression of several matrix metallopeptidases—enzymes that are capable of extracellular matrix degradation—in a NFκB-dependent manner, suggesting that TERT has a NFκB-mediated role in cancer cell invasion and metastasis [112].

Another possible TERT transcription regulation activity that supports tumor metastasis is promoting epithelial-to-mesenchymal transition (EMT). In their 2015 study using HCT116 and SW480 colon cancer cell lines, the Yang lab found that by physically interacting with the transcriptional repressor zinc finger E box-binding homeobox 1 (ZEB1), TERT downregulates the expression of E-cadherin, a tumor suppressor whose loss-of-function drives EMT [113]. Accordingly, ectopic TERT expression enhances cancer cell migration and metastasis both in vitro and in vivo [113]. Upon ZEB1-KD, E-cadherin expression is restored in HT116 and SW480 cells that overexpress TERT, which correlates with a decrease in cell migration as the cells adopt an epithelial-like morphology [113]. These findings implicate the importance of the TERT-ZEB1 complex in tumor metastasis, at least in colon cancer.

Transcription of tRNA-encoding genes is also under TERT regulation. In their 2016 study using five cancer cell lines of four different origins and two embryonic stem cell lines, the Tergaonkar lab observed that TERT expression level is proportional to the rates of cell proliferation and protein synthesis, and that TERT is particularly enriched at genomic regions corresponding to RNA polymerase III (pol III)-driven tRNA genes in a TERC-independent manner [114]. Luciferase reporter assays and co-immunoprecipitation analysis indicate that TERT activates tRNA promoters via its physical interaction with RNA pol III 32 kDa subunit (RPC32) [114]. Thus, TERT pro-proliferative effect in cancer may in part come from enhancing tRNA expression, which in turn expands the translational capacity of malignant cells.

Angiogenesis is another oncogenic process that may rely on TERT role as a transcription regulator. In 2016, the Cong lab discovered that TERT can interact with transcription factor Sp1 at the promoter region of vascular endothelial growth factor (VEGF)—a potent mitogen, and accordingly, recombinant TERT increases the expression level of VEGF in human umbilical vein endothelial cells (HUVECs) [115]. When these cells were plated on Matrigel, the investigators observed vascular tube formation that was susceptible to mithramycin—a Sp1 inhibitor, but not to pyrrolidine dithiocarbamate—a NFκB inhibitor, which indicates that TERT promotes VEGF expression primarily via Sp1 [115]. Additionally, quantitative PCR analysis reveals that TERT expression only correlates positively with VEGF out of 23 known angiogenesis-related genes [115]. Taken together, these findings identify Sp1 and VEGF as key players in TERT angiogenesis function.

## 5. Conclusions

The prevalence of TERT upregulation in cancer implicates that it has pro-malignant activities in addition to telomere maintenance. Many of TERT interacting partners directly regulate its functions, and thus, research into these species is necessary for a better understanding of TERT contributions to cancer development. While some proteins have well-defined roles in telomerase biology, others are still poorly understood due to the lack of follow-up evaluations after the initial finding, or because they are discovered recently. Furthermore, independent research groups using different experimental models and/or cell lines to assess TERT interacting proteins have given rise to contradicting conclusions. Nevertheless, such disparities highlight the dynamic nature of telomerase biogenesis and activities, as well as the prospect that tumors of different origins exploit TERT functions differently.

Apart from the diversity inherent in tumor biology, studies of TERT interacting proteins must also take into account the technical caveats of the PPI methods in use during data analysis. As TERT is low in abundance even in the context of cancer [116], some assays such as co-immunoprecipitation often require the non-physiological overexpression of its recombinant forms, which may lead to false positives. Conversely, the stringent conditions of complex purification may displace functionally significant proteins with weak or transient TERT-binding capacity, resulting in false negatives. Moreover, because most screening systems are antibody-based, investigators are at risk of missing novel entities for which no antibody is available and/or TERT association has not been predicted. To reveal the entire scope of TERT interaction network, complementary methods that allow detection of transient interactions—for instance, proximity labeling approaches—should be adopted. Overall, although understanding of TERT and its binding partners has certainly advanced over the last 20 years, many knowledge gaps still exist, such as the whole spectrum of TERT non-telomeric activities, and the extent to which different TERT activities are context-dependent. Therefore, more work remains to be done before TERT implications in cancer can be fully comprehended.

## Figures and Tables

**Table 1:** Detailed descriptions of TERT interacting proteins and their TERT-related functions. TERT binding partners without mapped interaction domain(s) are highlighted in gray.

	Protein(s)	TERT-Related Function(s)	Reference(s)
**Participate in TERT nuclear localization**	HSP90—p23	Chaperones, facilitate TERT nuclear import via microtubules, protect TERT from degradation in cytoplasm	[24,33]
	FKBP52	Immunophilin, facilitates TERT nuclear import via microtubules	[25]
	Importin α	Karyopherin, facilitates TERT nuclear import via nuclear pores	[28]
	Ran	GTPase, facilitates TERT nuclear import (and export) via nuclear pores	[28]
	14-3-3	Phosphoprotein-binding protein, promotes TERT nuclear localization by impairing TERT-CRM1 interaction, may participate in TERT anti-apoptotic function in the mitochondria	[30,31]
	AKT	Kinase, promotes TERT nuclear import via importin α mechanism by phosphorylating residue S227	[28]
	PP2A	Phosphatase whose activity antagonizes TERT nuclear import, may mediate TERT—14-3-3 interaction	[29]
**Participate in TERT nuclear export**	CRM1	Karyopherin, facilitates TERT nuclear export via nuclear pores	[30]
	Src	Kinase, promotes TERT nuclear export by phosphorylating residue Y707	[36]
	Shp2	Phosphatase, prevents TERT nuclear export by dephosphorylating residue Y707	[37]
**Participate in TERT degradation in cytoplasm**	MKRN1	Ubiquitin ligase, facilitates TERT degradation via the ubiquitin-proteasome pathway	[32]
	HSP70—CHIP	Chaperones, promote TERT degradation by enhancing TERT-MKRN1 interaction	[33]
**Interact with TERT in PML bodies**	PML IV	PML protein isoform 4, negative regulator of telomerase activity	[58]
**Interact with TERT in the nucleolus**	PINX1	Tumor suppressor, negative regulator of telomerase activity	[59,60]
	NPM	Phosphoprotein, positive regulator of telomerase activity	[61,62]
	MCRS2	RNA-binding protein, negative regulator of telomerase activity	[63]
	NCL	Phosphoprotein, facilitates TERT nucleolar localization	[65]
	Pontin, reptin, NVL2	ATPases, positive regulators of telomerase activity	[67,68]
	NAT10, GNL3L	NTPases, regulate telomerase activity in a context-/cell cycle-dependent manner	[69]
	PES1	Positive regulator of telomerase activity, may participate in TERT pro-proliferative function	[70]
**Interact with TERT in Cajal bodies**	TCAB1	Chaperone, facilitates (TERC-mediated) TERT localization in Cajal bodies, delivers telomerase to telomeres during catalysis	[43,44,45,79,80]
	Coilin, SMN	Constituents of Cajal bodies, may regulate telomerase activity in a context-dependent manner	[51,71,72,73]
	A2/B1, A18	RNA-binding proteins, positive regulators of telomerase activity	[76,77,78]
**Interact with TERT at telomeres**	TPP1	Shelterin subunit, directly tethers telomerase to telomeric DNA during catalysis, forms a dimer with POT1 which regulates telomerase activity in a context-/cell cycle-dependent manner	[84,85,89,90,91,92,93]
	KIP	Calcium-binding protein, tethers telomerase to shelterin subunit TRF2 during catalysis	[86,87]
**Participate in TERT non-telomeric activities**	p53	Tumor suppressor, contributes to TERT anti-apoptotic and pro-proliferative effects	[99,100]
	PARP	DNA damage repair protein, forms a ternary complex with TERT and p53	[99,101]
	MCL1, BCL-xL	Anti-apoptosis proteins, interact with TERT in the mitochondria	[102]
	BRG1	Transcription factor, engages TERT at promoter region of Wnt pathway target genes	[103]
	NFκB p65	Transcription factor, engages TERT at promoter region of selective NFκB target genes	[111]
	ZEB1	Transcription repressor, engages TERT at promoter region of E-cadherin gene	[113]
	RPC32	RNA polymerase III subunit, engages TERT at promoter regions of tRNA genes	[114]
	Sp1	Transcription factor, engages TERT at promoter region of VEGF gene	[115]

HSP90—heat shock protein 90; FKBP52—FK506-binding protein 52; PP2A—serine/threonine-protein phosphatase 2A; CRM1—chromosome region maintenance 1 protein homolog; MKRN1—E3 ubiquitin-protein ligase makorin-1; HSP70—heat shock protein 70; CHIP—carboxyl-terminus of HSP70 interacting protein; PML—promyelocytic leukemia protein; PINX1PIN2/TERF1—interacting telomerase inhibitor 1; NPM—nucleophosmin; MCRS2—microspherule protein 2; NCL—nucleolin; NVL2—nuclear valosin-containing protein-like 2; NAT10—N-acetyltransferase 10; GNL3L—guanine nucleotide-binding protein-like 3-like protein; PES1—Pescadillo homolog; TCAB1—telomerase Cajal body protein 1; SMN—survival motor neuron protein; TPP1—tripeptidyl-peptidase 1; POT1—protection of telomeres protein 1; TRF2—telomeric repeat-binding factor 2; PARP—poly [ADP-ribose] polymerase; MCL1—induced myeloid leukemia cell differentiation protein MCL1; BCL-xLBCL2-associated agonist of cell death; ZEB1—zinc finger E box-binding homeobox 1; RPC32—RNA pol III 32 kDa subunit; VEGF—vascular endothelial growth factor.
